# Complete mitochondrial genome of *Benthodytes marianensis* (Holothuroidea: Elasipodida: Psychropotidae): Insight into deep sea adaptation in the sea cucumber

**DOI:** 10.1371/journal.pone.0208051

**Published:** 2018-11-30

**Authors:** Wendan Mu, Jun Liu, Haibin Zhang

**Affiliations:** 1 Institute of Deep-sea Science and Engineering, Chinese Academy of Sciences, Sanya, China; 2 University of Chinese Academy of Sciences, Beijing, China; Natural History Museum of London, UNITED KINGDOM

## Abstract

Complete mitochondrial genomes play important roles in studying genome evolution, phylogenetic relationships, and species identification. Sea cucumbers (Holothuroidea) are ecologically important and diverse members, living from the shallow waters to the hadal trench. In this study, we present the mitochondrial genome sequence of the sea cucumber *Benthodytes marianensis* collected from the Mariana Trench. To our knowledge, this is the first reported mitochondrial genome from the genus *Benthodytes*. This complete mitochondrial genome is 17567 bp in length and consists of 13 protein-coding genes, two ribosomal RNA genes and 22 transfer RNA genes (duplication of two tRNAs: *trnL* and *trnS*). Most of these genes are coded on the positive strand except for one protein-coding gene (*nad6*) and five tRNA genes which are coded on the negative strand. Two putative control regions (CRs) have been found in the *B*. *marianensis* mitogenome. We compared the order of genes from the 10 available holothurian mitogenomes and found a novel gene arrangement in *B*. *marianensis*. Phylogenetic analysis revealed that *B*. *marianensis* clustered with *Peniagone* sp. YYH-2013, forming the deep-sea Elasipodida clade. Positive selection analysis showed that eleven residues (24 S, 45 S, 185 S, 201 G, 211 F and 313 N in *nad2*; 108 S, 114 S, 322 C, 400 T and 427 S in *nad4*) were positively selected sites with high posterior probabilities. We predict that *nad2* and *nad4* may be the important candidate genes for the further investigation of the adaptation of *B*. *marianensis* to the deep-sea environment.

## Introduction

Holothuroids, also known as sea cucumbers, are an abundant and diverse group, which are one of the five extant echinoderm classes. It includes more than 1400 species around the world [1]. They are present in diverse marine environments ranging from the shallow waters to the deepest oceanic trench [2]. Despite their diversity and abundance, our understanding of the higher-level phylogenetic relationships and evolution of Holothuroidea remains limited [1,3,4]. Multiple incongruences have been found between morphology based taxonomic systems [4,5] and molecular systematic reports [3,6,7].

Complete mitochondrial genomes not only provide information about individual genes, but also offer a broad range of genome-level characteristics which are useful for studying genome evolution, phylogenetic relationships, and species identification [8,9,10]. Therefore, mitogenomes have become more popular in recent years. Up to now, the complete mitogenomes have been reported in many marine organisms, such as shellfish [11,12,13], sea lily [14,15], sea urchin [16,17,18], starfish [19,20,21], pipefish [22], and crab [23].

In most metazoans, the mitochondrial genome is a small and closed-circular DNA molecule (between 14 and 18 kb in length), which generally contains 37 genes: 13 protein-coding genes (PCGs), 2 ribosomal RNA genes (rRNAs), and 22 transfer RNA genes (tRNAs) [24,25]. All 13 protein-coding genes play key roles in oxygen usage and energy metabolism [24,26]. Despite strong functional constraints, mitochondrial DNA may be subject to positive directional selection in response to pressures from extreme harsh environments [27]. In recent years, mitochondrial DNA analyses have demonstrated the existence of adaptive evolution in the cytochrome *c* oxidase genes [28,29], the cytochrome *b* gene [30], the NADH dehydrogenase genes [26,31,32], and the ATP synthase genes [31–33]. The mitogenomes of most metazoans studied have only one control region, which is involved in the initiation and regulation of transcription and replication of animal mitogenome [34]. However, the mitogenomes from some animals have two separate control regions with identical or highly similar nucleotide sequences [35–39].

Deep-sea (2,000–6,500 m) environments cover approximately 66% of global sea-floor area, and more than 50% of the planet’s surface [40,41]. The deep-sea is recognized as an extremely harsh environment [42]. The organisms living there survive with low food resources, low temperature, high hydrostatic pressure and constant darkness [42,43]. To evaluate the variation in deep-sea sea cucumber mitogenome features and their potential molecular mechanisms of adaptation to the deep-sea environment, we sequenced the complete mitochondrial genome of *Benthodytes marianensis* [44], which was collected from the Mariana Trench at 5567 meters depth. The order Elasipodida is a true deep water form and was erected by Théel [45], who reported Holothurioidea species collected during the H.M.S. Challenger Expedition.

There are several complete mitogenomes of sea cucumbers have been sequenced in recent years [46–50]. By using several complete mitogenomes, Sun et al. [49] concluded that three color variants of sea cucumber belong to a single species *Apostichopus japonicus*. Fan et al. [50] found a novel gene arrangement in the mitochondrial DNA of *Stichopus horrens*. However, few mitochondrial data of deep sea species has been reported. In the present study, we described the complete mitochondrial genome of *B*. *marianensis*, and identified its base composition, codon usage, gene arrangement, and phylogenetic relationships. To understand the adaptive evolution of mitochondrial genes, positive selection analysis was also conducted.

## Materials and methods

### Ethics statement

No specific permits were required for the sea cucumber collected in this study. The sampling locations were not privately owned or protected in any way and the collection did not involve endangered or protected species.

### Sample collection and DNA extraction

The specimen was collected at 5556 meters depth by the deep-sea Human Occupied Vehicle (HOV) “Jiao Long” during an expedition at the Mariana Trench (11°47.9757' N, 142°6.8535' E), on June, 2016. The sample was dissected and the muscle tissue samples were preserved in 95% ethanol or RNA later separately. Total genomic DNA was extracted from ethanol preserved tissue using TIANGEN marine animal DNA kit (TIANGEN, China).

### PCR amplification and sequencing

Four short fragments of *cox1*, *cox3*, *cob*, and *16S* were amplified with the primers COIurF1+COIurR2 [51], cox3F+cox3R [52], cobF424+cobR876 [53], and 16SarL+16SbrH [52]. In addition, partial sequence of *cox2*, *nad4L*, *nad4*, *nad5*, *nad1* and *nad2* were amplified with the degenerate primers designed in this study based on conserved regions of other sea cucumbers on GenBank. The remaining gaps were amplified with the species-specific primers designed according to the obtained sequences. Subsequently, the whole mitogenome was amplified based on ten pairs of primers (see S1 Table).

PCR reactions were performed with a gradient machine (Applied Biosystems Veriti Thermal Cycler) using TaKaRa LA Taq polymerase. PCR cycling was set up with an initial denaturation step at 94°C for 5 min, followed by 35 cycles comprising denaturation at 94°C for 30 sec, annealing at 45–62°C for 1 min (see S1 Table), and extension at 72°C for 1~4 min depending on the expected length of the PCR products. The process was completed with a final extension at 72°C for 10 min. The PCR products were directly purified with Gel Extraction Kit (Omega Bio-Tek) and sequenced in both directions using the PCR primers with ABI 3730x1 DNA Analyzer (Applied Biosystems Inc.).

### Sequence analysis and gene annotation

Raw sequences were first processed using Phred with the quality score 20 and assembled in Phrap with default parameters [54,55]. Then, all assemblies and sequence quality were verified manually in Consed [56]. DOGMA (http://dogma.ccbb.utexas.edu/) [57], ORFfinder (http://www.ncbi.nlm.nih.gov/projects/gorf/orfig.cgi) and BLAST (http://blast.ncbi.nlm.nih.gov/Blast.cgi) were used to identify protein encoding genes. The tRNA genes were identified by the program tRNAscan-SE 1.21 (http://lowelab.ucsc.edu/tRNAscan-SE/) [58] and MITOS webserver (http://mitos.bioinf.uni-leipzig.de/index.py) [59]. Secondary structures for tRNAs were drawn using MITOS webserver. The locations of the rRNA genes were determined based on alignments with the other holothuroid mitogenomes. Codon usage analysis was estimated with MEGA 5.0 [60]. The mitochondrial gene map was drawn with GenomeVx [61]. The AT skew = [A-T] / [A+T] and GC skew = [G-C] / [G+C] were used to describe the base composition difference between different class of Echinodermata [62].

### Phylogenetic analysis

Thirteen echinoderm mitogenomes including the one obtained in this study were used for phylogenetic analysis. All mtDNA sequences used are listed in S2 Table. *Strongylocentrotus purpuratus* and *Paracentrotus lividus* (Echinoidea) were rooted as the outgroup. Multiple alignments of the thirteen-partitioned amino acid sequences of protein-coding genes were conducted using MAFFT v7.037b [63]. Ambiguously aligned regions were removed using Gblocks ver. 0.91b [64] with the default option. The best-fit substitution models for each data partition were selected by ProtTest version 3.4 [65]. The information of alignment length and amino acid substitution models applied to each partition gene were listed in S3 Table.

Two phylogenetic reconstruction approaches were performed including Maximum Likelihood (ML) and Bayesian Inference (BI). ML analysis was conducted using RAxMLGUI v1.5b1 [66] applying the settings “ML + rapid bootstrap”, 1000 replicates, with the best-fit models as listed in S3 Table. Bayesian analysis was conducted using MrBayes v3.2.6 [67] applying the best fit models to each partition (S3 Table). Analyses had two parallel runs with four chains each (three hot and one cold) and were carried out for 5,000,000 generations (sampling every 100 generations). The two independent runs were checked with Tracer 1.6 [68]. After omitting the first 25,000 “burn in” trees, the remaining 25,000 sampled trees were used to estimate the 50% majority-rule consensus tree and the Bayesian posterior probabilities (PP).

### Positive selection analysis

The CODEML program from pamlX package [69,70] was used to identify selection. All the models corrected the average nucleotide frequencies at the three codon positions (CodonFreq = 2). The “one-ratios” model (model 0), “free-ratios” model (model 1) and “two-ratios” model were used in the combined dataset of 13 protein-coding genes to indicate that selective pressure differed between *B*. *marianensis* and the other ten sea cucumbers. The two branch sit models (A and A-null) were used to test whether these genes have experienced positive selection at some amino acids. Bayes Empirical Bayes (BEB) [71] analysis was used to calculate the posterior probabilities of the positively selected sites.

## Results and discussion

### Mitogenome content and gene organization

**Fig 1:**
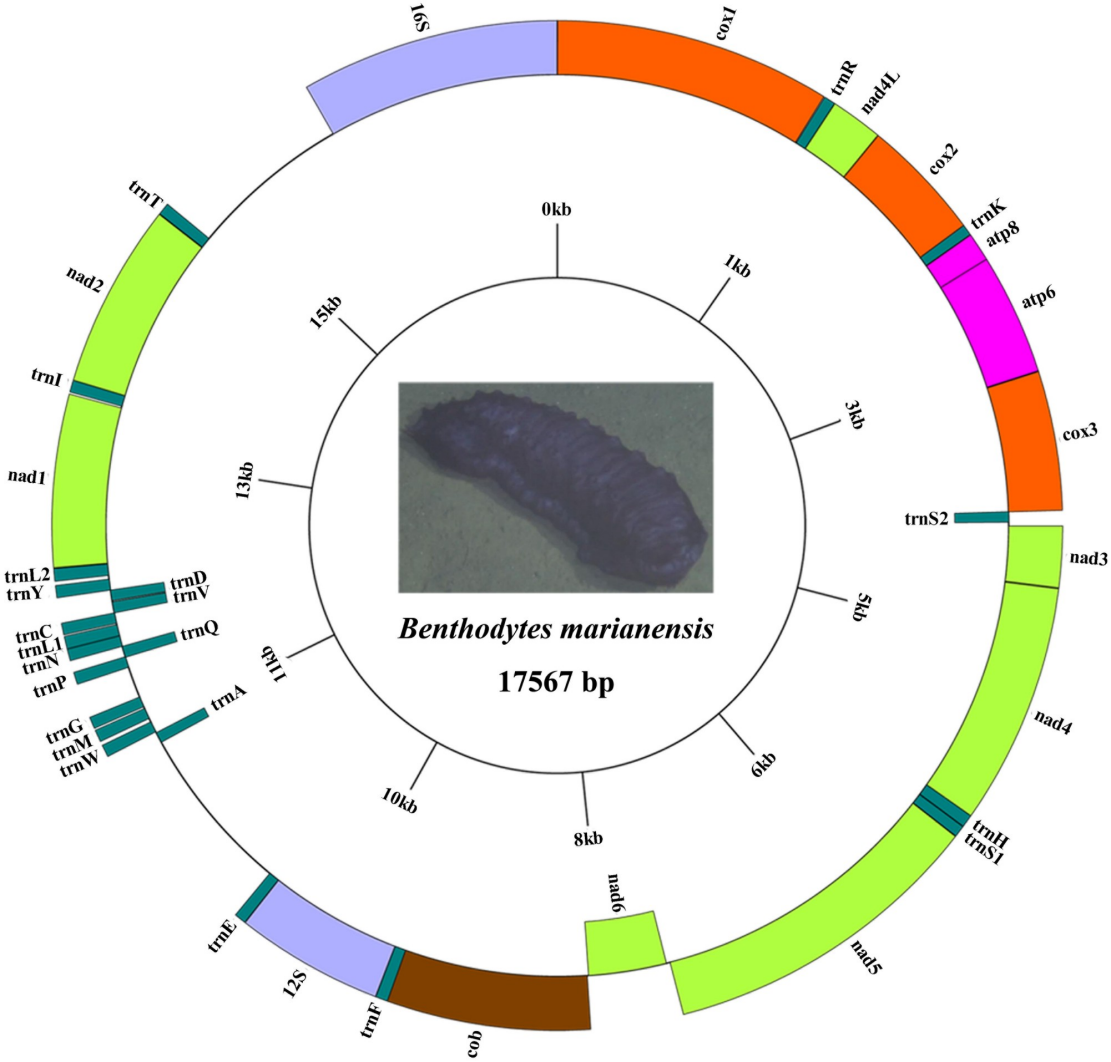
Genome map and annotation of *Benthodytes marianensis* complete mitochondrial genome. Genes out of the circle encode on positive strand (direction 5’→3’) and genes inner of the circle encode on negative strand (direction 3’→5’). Genes for protein coding genes and rRNAs are shown with standard abbreviation. Genes for tRNAs are designated by a single letter for the corresponding amino acid with two leucine tRNAs and two serine tRNAs differentiated by numerals.

The mitogenome of the *B*. *marianensis* described in the present study is a 17567 bp circular molecule (Fig 1) with a nucleotide composition of 32.33% A, 17.83% C, 12.93% G, 36.91% T bases in the positive strand. The genome encodes 37 genes including 13 protein-coding genes (PCGs), 2 rRNA genes, and 22 tRNA genes (two tRNAs are duplicated: *trnL* and *trnS*). Six genes are encoded on the negative strand, while the other 31 are encoded on the positive strand (Fig 1 and S4 Table). The complete mitochondrial DNA sequence has been deposited in the GenBank (accession no: **MH208310**).

A chi-square test with 1 d. f. demonstrated that the A+T content of *B*. *marianensis* mitogenome is significantly different from other holothurians (p < 0.01) (Table 1). This significant difference was also found with mtDNA composition of *Peniagone* sp. YYH-2013 (KF915304). Bohlin et al. [72] suggested that AT content is an indicator of relative investment in nucleotides and amino acids. Chen et al. [73] concluded that A+T/U base pairs are cheaper than G+C pairs, and this trade-off means that low-GC genomes spent proportionally less energy in nucleotide production than high-GC genomes. The size of holothurian mitogenomes range from 15507 bp (in *Peniagone* sp. YYH-2013) to 17567 bp (*B*. *marianensis*) (Table 1). The larger size of *B*. *marianensis*’s mitogenome is the result of the two putative control regions (CRs) in this species.

**Table 1:** Genomic characteristics of Holothuroidea mtDNAs.

Species	Accession number	Genome		Protein-coding genes		*16S* gene		*12S* gene		tRNAs		Non-coding regions		Largest non-coding regions		Reference
		Length (bp)	A+T (%)	Length (bp)	A+T (%)	Length (bp)	A+T (%)	Length (bp)	A+T (%)	Length (bp)	A+T (%)	Length (bp)	A+T (%)	Length (bp)	A+T (%)	
*Apostichopus japonicus*	NC_012616	16099	61.96	11387	61.45	1562	62.48	826	61.74	1512	62.30	915	66.89	452	59.73	[49]
*Benthodytes marianensis*	MH208310	17567	69.24	11400	67.81	1452	71.07	834	66.43	1506	70.92	2428	75.04	1062	74.86	This study
*Holothuria forskali*	NC_013884	15841	62.22	11365	62.13	1577	61.45	830	61.20	1440	60.62	577	71.75	421	67.70	[46]
*Holothuria scabra*	NC_027086	15779	59.74	11360	59.68	1532	60.77	820	57.44	1515	58.35	566	65.72	456	62.06	[48]
*Cucumaria miniata*	NC_005929	17538	63.83	11339	62.25	1319	64.90	881	65.27	1505	62.25	2500	69.08	1159	72.82	[47]
*Parastichopus nigripunctatus*	NC_013432	16112	61.82	11308	61.45	1564	61.70	826	61.62	1527	62.15	848	68.16	456	59.65	Sasaki and Hamaguchi, Unpublished
*Parastichopus californicus*	NC_026727	16727	61.40	11385	60.61	1564	62.21	824	61.89	1526	62.39	1466	65.83	472	60.38	Liu, Unpublished
*Parastichopus parvimensis*	NC_029699	16120	61.69	11360	61.21	1606	62.52	828	61.96	1521	61.93	860	66.63	438	60.50	Zhang et al., Unpublished
*Peniagone* sp. YYH-2013	KF915304	15507	73.41	11346	72.59	1461	73.85	830	71.08	1412	76.77	547	85.19	391	81.33	Huo et al., Unpublished
*Stichopus horrens*	NC_014454	16257	60.11	11397	59.82	1642	61.02	820	58.41	1520	62.70	903	59.91	676	58.14	[50]
*Stichopus* sp. SF-2010	NC_014452	16257	60.23	11384	59.80	1653	61.65	810	59.51	1531	62.12	902	60.98	674	58.61	Fan and Hu, Unpublished

### Base composition and AT/GC-skew of mtDNA in Echinodermata

**Fig 2:**
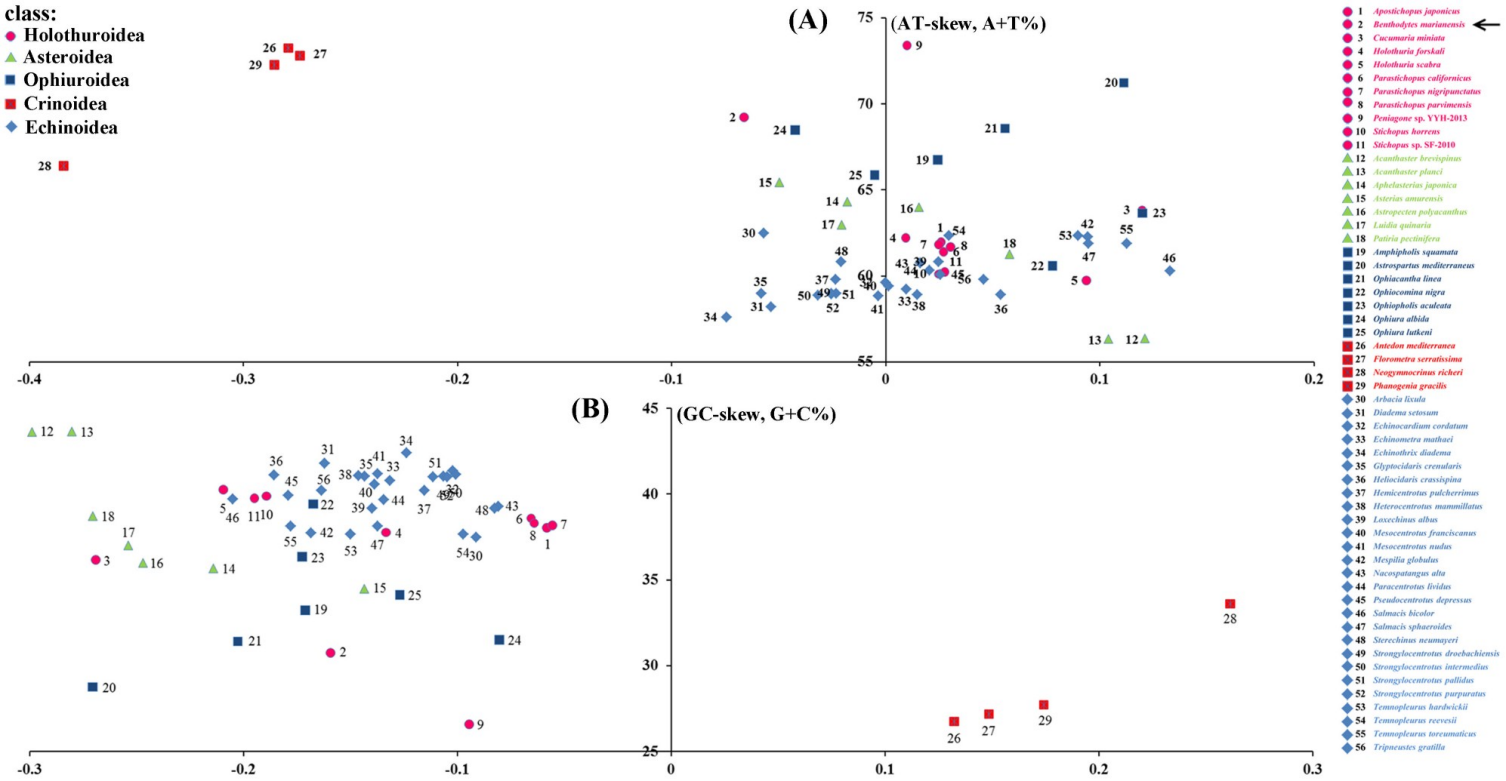
A+T% vs AT skew (a) and G+C% vs GC skew (b) in the 56 echinoderm mitochondrial genomes. Values are calculated on the positive strand for the full length of the mitogenomes. The X-axis provides the skew values, while the Y axis provides the A+T/ G+C values. Numbers and names of species are colored according to their taxonomic placement (see more information of these species in S5 Table).

A+T/G+C content and A-T/G-C skew of the positive strand are shown in Fig 2 for *B*. *marianensis* and other 55 echinoderm species. There was considerable variation in A+T content among different species, ranging from 56.34% (13: *Acanthaster planci*) to 73.41% (9: *Peniagone* sp. YYH-2013). In different classes within the phylum Echinodermata, the A+T% values showed different ranges: Holothuroidea (11 species), 59.74–73.41%, average: 63.24±4.26%; Asteroidea (7 species), 56.34–65.45%, average: 61.53±3.76%; Ophiuroidea (7 species), 60.58–71.24%, average: 66.45±3.52%; Crinoidea (4 species), 66.39–73.26%, average: 71.19±3.22%; Echinoidea (27 species), 57.61–62.50%, average: 60.05±1.40%. The average of A+T% value for all 56 mitogenomes is 62.46±4.28%.

The echinoderm AT-skews varied from -0.3839 (*Neogymnocrinus richeri*) to 0.1329 (*Salmacis bicolor*) with the *B*. *marianensis* mitogenome exhibiting an almost balanced composition for A and T (AT-skew = -0.0661). The GC-skews of echinoderms varied from -0.2989 (*Acanthaster brevispinus*) to 0.2615 (*Neogymnocrinus richeri*) with the *B*. *marianensis* mitogenome being strongly skewed away from G in favor of C (GC-skew = -0.1593). The AT-skew values exhibited different ranges among five echinoderm classes: Holothuroidea [-0.0661–0.1198], Asteroidea [-0.0497–0.1210], Ophiuroidea [-0.0423–0.1199], Crinoidea [-0.3839-(-0.2736)], and Echinoidea [-0.0743–0.1329] (Fig 2).

In general, the A+T content from high to low are: Crinoidea > Ophiuroidea > Asteroidea > Holothuroidea (except the two deep-sea species) > Echinoidea (Fig 2). When comparing the skew values of the five classes, we found that the AT/GC-skew values of the class Crinoidea was lower than the other four classes.

### Overlapping and non-coding regions

The mitogenome of *B*. *marianensis* contains six overlapping regions with a total length of 53 bp. The four overlapping regions are between a protein coding gene and a tRNA (*cox2*/*trnK*, *cox3*/*trnS*_*2*_, *nad4*/*trnH*, and *cob*/*trnF*), one protein coding gene to protein coding gene (*atp8*/*atp6*), and one tRNA to tRNA (*trnP*/*trnQ*). The individual length of these overlaps varies from 2 bp to 20 bp, and the longest one existed between *cob* and *trnF* (S4 Table).

The 21 non-coding regions (NCRs) were found with a length varying from 1 bp to 1060 bp (S4 Table). Two putative control regions (CRs) have been identified in *B*. *marianensis* mitogenome: CR1 was 1060 bp (A+T% = 74.91), flanked by *trnE* and *trnA*, and CR2 was 1010 bp (A+T% = 74.06), flanked by *trnT* and *16S*. Sequence similarity of CR1 and CR2 was 88.6%. Compared with other ten sea cucumber mitogenomes, only one species (*Cucumaria miniata*) had duplicated CRs, whereas nine others had only one CR. The two putative control regions of *C*. *miniata* have been described as having of 83.5% similarity: one being 405 bp long and the other 452 bp [36,47]. Duplicated CRs have also been found in the mitogenomes of birds [39,74,75], snakes [35,76], fishes [38,77,78,79], sea firefly [80], ticks [81], and thrips [82]. Shao et al. [81] found that tandem duplication is the most possible mechanism for the generation of duplicate CRs in mitogenome of plague thrips. Shi et al. [83] inferred that the duplicate CRs were generated from a dimer molecule formed by two monomers linked head-to-tail in the mitogenome of *Crossorhombus azureus*. Then one of the two promoters lost function and the other became the new CR. Li et al. [79] hypothesized that the duplicate CRs of the bothids were generated in the similar dimerization model.

How these duplicate CRs were generated and how did mitogenome with duplicate CRs evolve in sea cucumbers are indeed interesting yet currently unresolved questions. Many researches support the notion that mitogenome with duplicate CRs may replicate more efficiently than mitogenome with a single CR [35,36,84]. Yet, as only two species of sea cucumbers have currently been shown to have duplicated CRs, additional sea cucumber mitogenomes are needed to clarify the mechanism forming this phenomenon.

### Protein-coding genes

**Fig 3:**
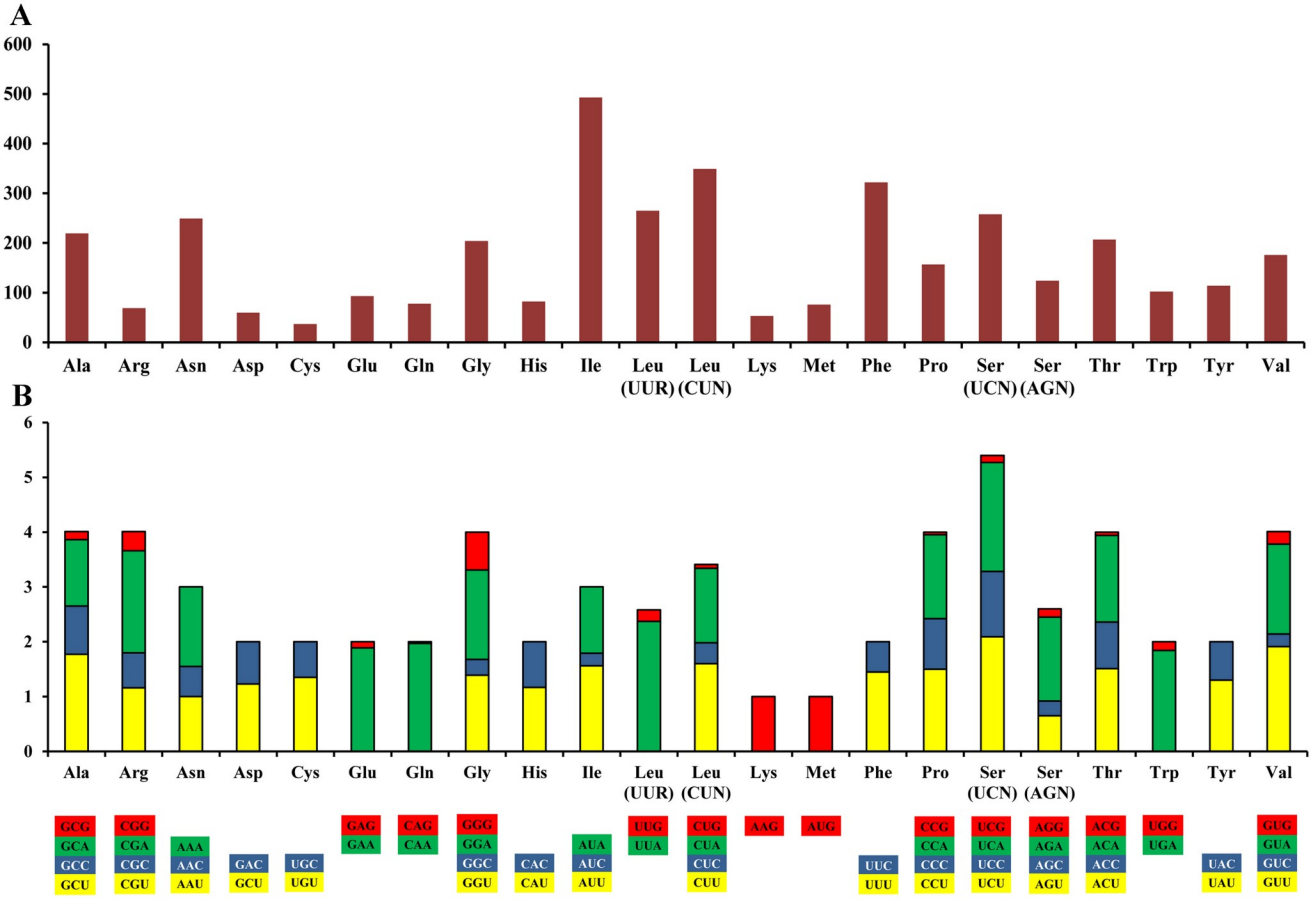
Codon usage (A) and the relative synonymous codon usage (RSCU) (B) of *Benthodytes marianensis* mitogenome. Numbers to the left refer to the total number of codons (A) and the RSCU value (B). Codon families are provided on the X-axis.

All PCGs were encoded by the positive strand, except for *nad6* that was encoded by the negative strand. This orientation has been observed in all of the holothurian mitogenomes published so far. The 13 PCGs initiate with the standard start codon ATN, which is typical for metazoan mitogenomes [34]. Most PCGs terminate with the stop codon TAA (10 out of 13), and three genes terminate with stop codon TAG. The total length of the PCGs sequences in *B*. *marianensis* is 11400 bp, and the A+T content is 67.81% (Table 1). The usage of amino acids and relative synonymous codon usage (RSCU) values in the PCGs of *B*. *marianensis* are summarized in Fig 3. The total number of codons (except stop codon) in *B*. *marianensis* are 3787. Among PCGs, isoleucine (13.02%) and cysteine (0.98%) are the most and the least used amino acids, respectively. The RSCU indicates the five most used codons: TTA (Leu), TCT (Ser), TCA (Ser), CAA (Gln) and GTT (Val) (Fig 3). Additionally, compared with other synonymous codons, the codons with A and T in the third position are always the most used. It is obvious that the A+T content of the third codon position (78.80%) is higher than the first (60.73%) and second positions (63.64%). This phenomenon has also been reported in many studies, such as in abalone, oyster, and scallop [11–13].

### Ribosomal RNA and transfer RNA genes

The boundaries of both the small (*12S*) and the large (*16S*) ribosomal genes were determined based on alignments with the other holothuroid mitogenomes. The *16S* and *12S* genes of *B*. *marianensis* are 1452 bp (A+T% = 71.07) and 834 bp (A+T% = 66.43) in length, respectively.

We analyzed the entire mitogenome sequence of *B*. *marianensis* and successfully identified 22 tRNA genes based on their potential secondary structures using the tRNAscan-SE [58] and MITOS webserver [59] (S1 Fig and S4 Table). The sequences of 22 tRNA genes range from 64 bp (*trnK*) to 73 bp (*trnH*). The two leucine and two serine tRNA genes were differentiated by their anticodon sequences. Twenty-one of these genes display a common clover-leaf secondary structure, and the remaining one absent dihydrouracil arm from *trnI*. Loss of the DHU arm from *trnI* has also been observed in other holothurians, such as *Holothuria scabra* [48].

### Phylogenetic analysis

**Fig 4:**
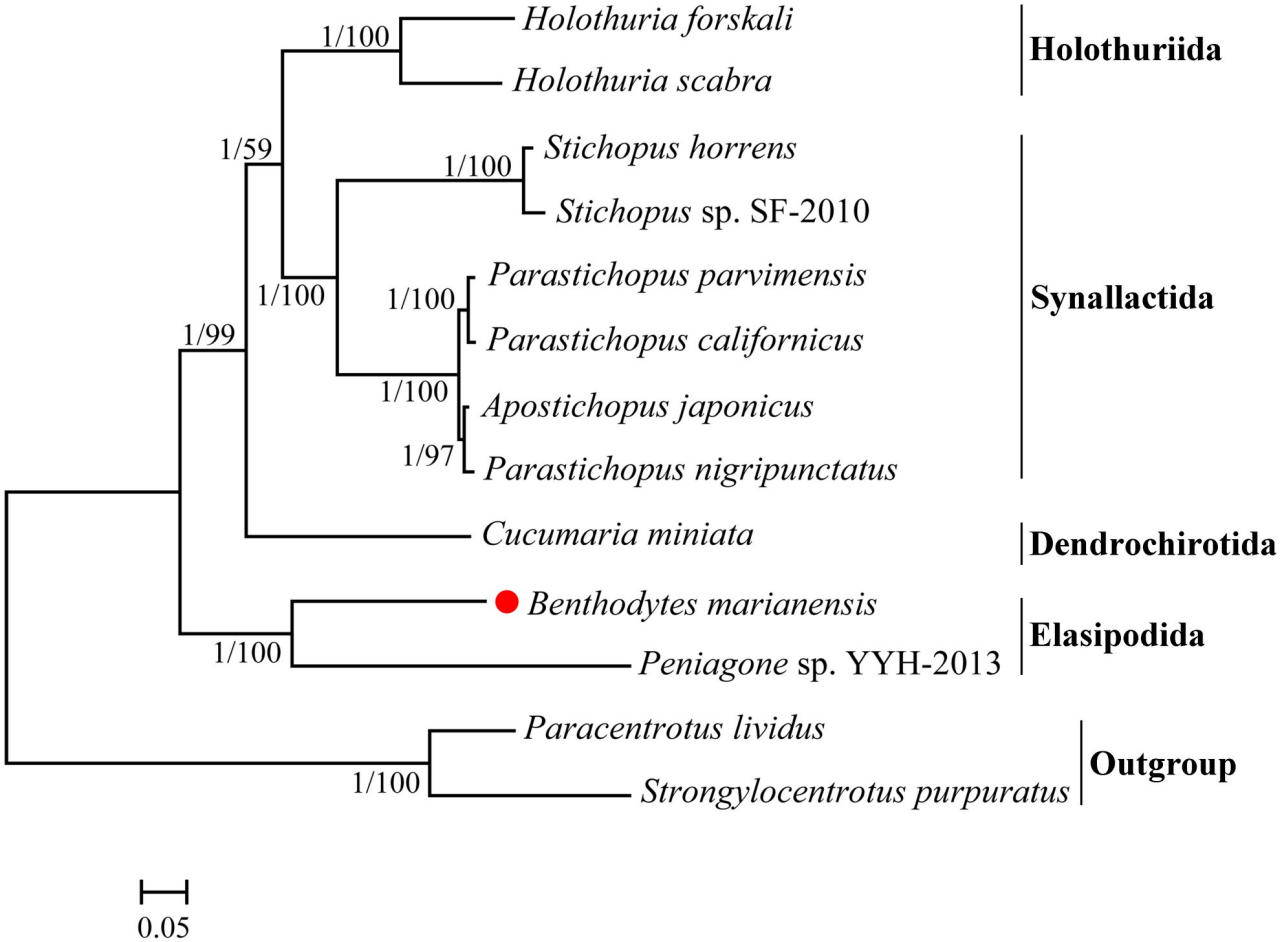
Phylogenetic trees based on the concatenated amino acid of 13 protein-coding genes. The branch lengths are determined with ML analysis. The *Strongylocentrotus purpuratus* and *Paracentrotus lividus* are used as outgroup. In BI and ML trees, the first number at each node is Bayesian posterior probability and the second number is the bootstrap probability of ML analyses. The red dot highlights the species sequenced in this study.

We performed the phylogenetic analysis by using 13 concatenated mitochondrial protein-coding gene amino acid sequences to provide evolutionary relationships among the 11, currently available, Holothuroidea species (Fig 4). Both Maximum Likelihood (ML) and Bayesian Inference (BI) analyses produced identical tree topologies with varying levels of support (Fig 4). Almost all the clades were strongly supported (i.e., posterior probabilities = 1 and bootstrap support = 100). The mitogenomic phylogenetic analyses showed that *B*. *marianensis* was clustered with *Peniagone* sp. YYH-2013 (posterior probabilities = 1 and bootstrap support = 100) in the Elasipodida clade. The order Elasipodida then clustered with Dendrochirotida, Holothuriida and Synallactida, successively. It revealed that Elasipodida was positioned at the base of the (Dendrochirotida + (Holothuriid + Synallactida)) clade.

This relationship between Elasipodida and other order of the holothuroids is also supported by previously published studies [7,85].

Taxonomic relationships among Elasipodida have varied over the years. There are four represented family-ranked taxa within Elasipodida: Elpidiidae, Laetmogonidae, Pelagothuriidae, and Psychropotidae. However, holothurians of the family Psychropotidae are amongst the least studied deep-sea holothuroids [86]. To better understand the relationship among them, further research on the diverse Elasipodida species is needed.

### Gene rearrangements

Gene order comparisons may act as a powerful tool for phylogenetic studies [87,88]. During the past twenty years, many studies on mitochondrial gene orders in echinoderms have been reported [1,14,15,24,46,89,90]. There are four possible mechanisms for genome rearrangement: inversion (reversals), transposition, reverse transposition and tandem duplication random losses (TDRLs) [14].

In the current study, a comparison of *B*. *marianensis* with the other 10 currently available Holothuroidea mitogenomes is shown in S2 Fig. Gene arrangements were mapped onto the phylogenetic trees in S2 Fig in order to get insights on the mechanisms of Holothuroids mitogenome rearrangements. All of the currently available Holothuroidea mitogenomes come from four orders: Dendrochirotida, Elasipodida, Holothuriida and Syanallactida. Gene order of the 13 PCGs in the 11 holothuroid species is identical, while several tRNAs have been translocated. The order of mtDNA genes of *Apostichopus japonicas*, *Parastichopus californicus*, *Parastichopus paruimensis*, *Parastichopus nigripunctatus*, *Holothuria forskali* and *Holothuria scabra* is completely identical to each other. Between *Stichopus horrens*, *Stichopus* sp. SF-2010 and the former six species, one tRNA gene rearrangement has been observed. *B*. *marianensis* and *Peniagone* sp. YYH-2013 only share four identical gene blocks: *cox1*-*R*-*nad4L*-*cox2*-*K*-*atp8*-*atp6*-*cox3*-*S2*-*nad3*-*nad4*-*H*-*S1*-*nad5*, *nad6*-*cob*-*F*-*12S*-*E*, *L2*-*nad1*-*I*-*nad2* and one small blocks *N*-*L*_*1*_. Comparing *B*. *marianensis* with *C*. *miniata* identifies 10 tRNA genes rearranged. These results are consistent to those earlier reports that tRNA genes may be among the most mobile elements in the mitogenome [36,50]. *B*. *marianensis* and *C*. *miniata* are the only two holothurian species known to date to possess two mitochondrial control regions (S2 Fig). Consequently, the gene arrangement of *B*. *marianensis* mitogenome is unique among the published arrangement of sea cucumber genes.

### Positive selection analysis

Because the colonization of deep-sea environments may affect the function of mitochondrial genes, we investigated the potential positive selection pressures in *Benthodytes*. Results for the selective pressure analyses are shown in Table 2. When the ω (d_N_/d_S_) ratios for the 13 concatenated mitochondrial protein-coding genes were compared between *B*. *marianensis* and the other nine shallow sea sea cucumbers, we failed to find a significant difference in their ω ratios (chi-square: *p* > 0.05), indicating the ω ratio of *B*. *marianensis* branch (ω1 = 0.05055) has no significantly difference with the other nine sea cucumbers (ω0 = 0.05179). However, when analyzing individual genes, we found that eleven residues were identified as positively selected sites with high posterior probabilities (BEB values > 95%) in *nad2* (24 S, 45 S, 185 S, 201 G, 211 F, 313 N), *nad4* (108 S, 114 S, 322 C, 400 T, 427 S), which suggested positive selection may exist in these amino acid sites (Table 2).

**Table 2:** Selective pressure analyses of the mitochondrial genes of sea cucumber.

	**Branch model**										
**Trees**	**Model**	**lnL**		**Estimates of parameters**			**Model compared**		**2ΔlnL**		**LRT *p*-value**
BI/ML	Model 1	-69624.717095					Model 1 versus Model 0		395.10903		0.00000
	Two-ratio	-69822.254097		ω0 = 0.05179 ω1 = 0.05055			Two ratio versus Model 0		0.03503		0.85160
	Model 0	-69822.271610		ω = 0.05168							
	**Branch site model**										
**Gene**	**Model**	**lnL**	**Estimates of parameters**					**Model compared**	**2ΔlnL**	**LRT *p*-value**	**Positive sites**
*nad2*	Model A	-6383.335554	site class	0	1	2a	2b	Model A versus Model A null	3.95842	0.04664	24 S 0.967*
			proportion	0.75555	0.11472	0.11262	0.01710				45 S 0.962*
			Background ω	0.03420	1.00000	0.03420	1.00000				185 S 0.963*
			Foreground ω	0.03420	1.00000	29.15832	29.15832				201 G 0.973*
	Model A null	-6385.314765									211 F 0.952*
											313 N 0.953*
*nad4*	Model A	-9195.375863	site class	0	1	2a	2b	Model A versus Model A null	6.30914	0.01201	108 S 0.974*
			proportion	0.78471	0.12231	0.08044	0.01254				114 S 0.956*
			Background ω	0.05564	1.00000	0.05564	1.00000				322 C 0.986*
			Foreground ω	0.05564	1.00000	5.34340	5.34340				400 T 0.966*
	Model A null	-9198.530434									427 S 0.976*

ω = d_N_/d_S_; Model 0: “one-ratios” model; Model 1: “free-ratios” model; Two-ratio: “two-ratios” model.

*posterior probability > 95%.

Similar results have been found in other deep-sea animals (e.g., sea anemones and alvinocaridid shrimp), where it was suggested this may be related to the adaptive evolution to the environment [31,32]. Under the deep-sea extreme harsh environment, survival may require a modified and adapted energy metabolism [32,42,43]. Subsequently, *nad2* and *nad4* are suggested to act as a proton pump [30,91], thus mutations in these proteins could influence the efficiency of proton pumping processes. In previous studies, the protein-coding genes of NADH dehydrogenase have been considered important in the adaptive evolution of the mammalian mitogenome [26,30,91,92]. Therefore, we predict that mitochondrial genes, specifically *nad2* and *nad4*, may play an important role in *B*. *marianensis*’s adaptation to the deep-sea environment.

## Conclusions

This study characterized the complete mitogenome of the deep-sea *B*. *marianensis*, which is 17567 bp in length and encodes 37 genes including 13 PCGs, 2 rRNA genes, and 22 tRNA genes on both strands. We analyzed the mitogenome features, AT/GC-skew, codon usage, gene arrangement, phylogenetic relationships, and positive selection of *B*. *marianensis*. Two putative control regions (CRs) have been found in the mitogenome of *B*. *marianensis*. Moreover, a novel gene arrangement is described here for the Holothuroidea mitochondrial genome. More information on the structure and function of the mitochondrial genomes from deep-sea sea cucumbers are needed to reveal their adaptation to the deep-sea environment. Our study provides a clue and establishes a necessary foundation for further study.

## Supporting information

S1 Table Primers used for amplifying and sequencing the mitogenome of *Benthodytes marianensis*. (DOCX)

S2 Table List of taxa used in the phylogenetic analysis. (DOCX)

S3 Table The information of alignment length and amino acid substitution models applied to each partition gene. (DOCX)

S4 Table Gene content of the *Benthodytes marianensis* mitogenome. (DOCX)

S5 Table Information concerning the echinoderm species with complete mitochondrial genome used in this study. (DOCX)

S1 Fig Putative secondary structures for the 22 transfer RNAs of the *Benthodytes marianensis* mitogenome. (TIF)

S2 Fig Comparison of mitochondrial gene arrangement in Holothuroidea. For the purpose of presentation, the circular mitogenomes are linearized at the boundary between *cox1* and *16S*. Genes and control regions are shown as boxes. Control regions are abbreviated as CR and are highlighted in red color. tRNA genes are named with their single letter amino acid abbreviations. Gene segments are not draw to scale.
(TIF)
