# Supplementary material for: Complete mitochondrial genome of Benthodytes marianensis (Holothuroidea: Elasipodida: Psychropotidae): Insight into deep sea adaptation in the sea cucumber
Source: PLoS One. 2018 Nov 30;13(11):e0208051. doi: 10.1371/journal.pone.0208051 (PMC6267960; doi:10.1371/journal.pone.0208051)
Supplement: S1 Table — (DOCX) [file pone.0208051.s001.docx]

**Supplementary Table 1: Primers used for amplifying and sequencing the mitogenome of *Benthodytes marianensis*.**

| Name | Sequence(5’-3’) | Region | Annealing temperature | Location | Reference |
| --- | --- | --- | --- | --- | --- |
| COIurF1 | ACTGCCCACGCCCTAGTAATGATATTTTTTATGGTRATGCC | *cox1* | 62℃ | 175-215 | [51] |
| COIurR2 | TCGTGTGTCTACGTCCATTCCTACTGTRAACATRTG | *cox1* |  | 874-909 |  |
| cox3F | TGGTGGCGAGATGTKKTNCGNGA | *cox3* | 45℃ | 3690-3712 | [52] |
| cox3R | ACWACGTCKACGAAGTGTCARTATCA | *cox3* |  | 4239-4264 |  |
| cobF424 | GGWTAYGTWYTWCCWTGRGGWCARAT | *cob* | 50℃ | 8988-9013 | [53] |
| cobR876 | GCRTAWGCRAAWARRAARTAYCAYTCWGG | *cob* |  | 9408-9436 |  |
| 16SarL | CGCCTGTTTATCAAAAACAT | *16S* | 55℃ | 16892-16912 | [52] |
| 16SbrH | CCGGTCTGAACTCAGATCACGT | *16S* |  | 17427-17448 |  |
| T1-F | GGGGTTGAAAGAGGAGCAGGTA | *cox1* | 55℃ | 349-370 | In this study |
| T1-R | GGCGGATAGGTTTAAGGTGAGG | *cox2* |  | 2355-2376 |  |
| T2-F | CTCCTTTGCCTTGAACTCCTTC | *nad4L* | 49℃ | 1713-1734 | In this study |
| T2-R | GTTGACCCGTAAATTCTATCTG | *cox3* |  | 4087-4108 |  |
| T3-F | GCAATGACTGCTGGTTTA | *cox3* | 48℃ | 3597-3614 | In this study |
| T3-R | AGAAGTGTTCTCGTGGAG | *nad4* |  | 6022-6039 |  |
| T4-F | CACTTCCTTCCCATTCGTTC | *nad3* | 52℃ | 4457-4476 | In this study |
| T4-R | GCTGATGCTTTGGCTTTCAC | *nad5* |  | 6750-6769 |  |
| T5-F | GCCCTAATTGCATATTCCTCAG | *nad4* | 50℃ | 5583-5604 | In this study |
| T5-R | GCAGACTTTCCTATTGCTCCTA | *nad5* |  | 6931-6952 |  |
| T6-F | TGTGAAAGCCAAAGCATCAG | *nad5* | 52℃ | 6749-6768 | In this study |
| T6-R | AACCAACGAAGGCTGTAAGA | *cob* |  | 8972-8991 |  |
| T7-F | TGACCTCCCAACTCCAAGCA | *cob* | 55℃ | 8657-8676 | In this study |
| T7-R | TAGTGCAAATGGGCCACCAG | *nad1* |  | 13599-13618 |  |
| T8-F | ATAAAGGAAACCCTAAAGCC | *nad1* | 48℃ | 13121-13140 | In this study |
| T8-R | TTAAAGTTGTGGGATCGTCA | *nad2* |  | 14931-14950 |  |
| T9-F | TTTGCTTGCTTATAGGGACT | *nad2* | 48℃ | 14023-14042 | In this study |
| T9-R | GGGCTTTAATGGACGAAC | *16S* |  | 17408-174025 |  |
| T10-F | AAGTTAAGCCTGCCCGGTGAT | *16S* | 55℃ | 16932-16952 | In this study |
| T10-R | CCAGCAAGCCCAAGGAAATG | *cox1* |  | 1285-1304 |  |
