# Supplementary material for: Complete mitochondrial genome of Benthodytes marianensis (Holothuroidea: Elasipodida: Psychropotidae): Insight into deep sea adaptation in the sea cucumber
Source: PLoS One. 2018 Nov 30;13(11):e0208051. doi: 10.1371/journal.pone.0208051 (PMC6267960; doi:10.1371/journal.pone.0208051)
Supplement: S2 Table — (DOCX) [file pone.0208051.s002.docx]

**Supplementary Table 2: List of taxa used in the phylogenetic analysis.**

| **Taxon** | **Classification** | **Accession Number** | **Reference** |
| --- | --- | --- | --- |
| **Echinoidea** |  |  |  |
| *Strongylocentrotus purpuratus* | Echinoidea; Euechinoidea; Echinacea; Echinoida; Strongylocentrotidae | NC_001453 | [16] |
| *Paracentrotus lividus* | Echinoidea; Euechinoidea; Echinacea; Echinoida; Echinidae | NC_001572 | [17] |
| **Holothuroidea** |  |  |  |
| *Apostichopus japonicus* | Holothuroidea; Aspidochirotacea; Synallactida; Stichopodidae | NC_012616 | [49] |
| *Benthodytes marianensis* | Holothuroidea; Aspidochirotacea; Elasipodida; Psychropotidae | MH208310 | This study |
| *Holothuria forskali* | Holothuroidea; Aspidochirotacea; Holothuriida; Holothuriidae | NC_013884 | [46] |
| *Holothuria scabra* | Holothuroidea; Aspidochirotacea; Holothuriida; Holothuriidae | NC_027086 | [48] |
| *Cucumaria miniata* | Holothuroidea; Dendrochirotacea; Dendrochirotida; Cucumariidae | NC_005929 | [47] |
| *Parastichopus nigripunctatus* | Holothuroidea; Aspidochirotacea; Synallactida; Stichopodidae | NC_013432 | Sasaki and Hamaguchi ,Unpublished |
| *Parastichopus californicus* | Holothuroidea; Aspidochirotacea; Synallactida; Stichopodidae | NC_026727 | Liu, Unpublished |
| *Parastichopus parvimensis* | Holothuroidea; Aspidochirotacea; Synallactida; Stichopodidae | NC_029699 | Zhang et al., Unpublished |
| *Peniagone* sp. YYH-2013 | Holothuroidea; Aspidochirotacea; Elasipodida; Elpidiidae | KF915304 | Huo et al., Unpublished |
| *Stichopus horrens* | Holothuroidea; Aspidochirotacea; Synallactida; Stichopodidae | NC_014454 | [50] |
| *Stichopus* sp. SF-2010 | Holothuroidea; Aspidochirotacea; Synallactida; Stichopodidae | NC_014452 | Fan and Hu, Unpublished |
