# Supplementary material for: Complete mitochondrial genome of Benthodytes marianensis (Holothuroidea: Elasipodida: Psychropotidae): Insight into deep sea adaptation in the sea cucumber
Source: PLoS One. 2018 Nov 30;13(11):e0208051. doi: 10.1371/journal.pone.0208051 (PMC6267960; doi:10.1371/journal.pone.0208051)
Supplement: S3 Table — (DOCX) [file pone.0208051.s003.docx]

| Partition | Alignment length | Substitution models |
| --- | --- | --- |
| *cox1* | 517 | MtREV+G |
| *cox2* | 229 | MtArt+G |
| *cox3* | 260 | MtArt+G |
| *cob* | 376 | MtArt+G |
| *nad1* | 317 | MtArt+G |
| *nad2* | 329 | MtArt+G+F |
| *nad3* | 106 | MtREV+G |
| *nad4* | 380 | CpREV+G+F |
| *nad4L* | 97 | MtArt+G |
| *nad5* | 582 | MtREV+I+G+F |
| *nad6* | 160 | MtArt+G |
| *atp6* | 221 | MtArt+G |
| *atp8* | 59 | MtArt+I+G+F |

**Supplementary Table 3: The information of alignment length and amino acid substitution models applied to each partition gene.**
