# Supplementary material for: Complete mitochondrial genome of Benthodytes marianensis (Holothuroidea: Elasipodida: Psychropotidae): Insight into deep sea adaptation in the sea cucumber
Source: PLoS One. 2018 Nov 30;13(11):e0208051. doi: 10.1371/journal.pone.0208051 (PMC6267960; doi:10.1371/journal.pone.0208051)
Supplement: S4 Table — (DOCX) [file pone.0208051.s004.docx]

**Supplementary Table 4: Gene content of the *Benthodytes marianensis* mitogenome.**

| Gene | Location | | Size | | Codon | | Anticodon | Intergenic nucleotide(bp) | Strand |
| --- | --- | --- | --- | --- | --- | --- | --- | --- | --- |
|  | Start | End | Nucleotide(bp) | Amino acid | Start | Stop |  |  |  |
| *cox1* | 1 | 1554 | 1554 | 517 | ATG | TAA |  | 0 | H |
| *trnR* | 1560 | 1625 | 66 |  |  |  | TCG | 5 | H |
| *nad4L* | 1626 | 1922 | 297 | 98 | ATT | TAA |  | 0 | H |
| *cox2* | 1923 | 2621 | 699 | 232 | ATG | TAG |  | 0 | H |
| *trnK* | 2612 | 2675 | 64 |  |  |  | CTT | -10 | H |
| *atp8* | 2677 | 2844 | 168 | 55 | ATG | TAA |  | 1 | H |
| *atp6* | 2838 | 3521 | 684 | 227 | ATG | TAA |  | -7 | H |
| *cox3* | 3525 | 4307 | 783 | 260 | ATG | TAA |  | 3 | H |
| *trnS_2_* | 4306 | 4371 | 66 |  |  |  | TGA | -2 | L |
| *nad3* | 4394 | 4738 | 345 | 114 | ATG | TAA |  | 22 | H |
| *nad4* | 4743 | 6113 | 1371 | 456 | ATG | TAG |  | 4 | H |
| *trnH* | 6104 | 6176 | 73 |  |  |  | GTG | -10 | H |
| *trnS_1_* | 6178 | 6244 | 67 |  |  |  | GCT | 1 | H |
| *nad5* | 6246 | 8078 | 1833 | 610 | ATG | TAA |  | 1 | H |
| *nad6* | 8101 | 8589 | 489 | 162 | ATG | TAG |  | 22 | L |
| *cob* | 8598 | 9761 | 1164 | 387 | ATG | TAA |  | 8 | H |
| *trnF* | 9742 | 9811 | 70 |  |  |  | GAA | -20 | H |
| *12S* | 9812 | 10645 | 834 |  |  |  |  | 0 | H |
| *trnE* | 10646 | 10713 | 68 |  |  |  | TTC | 0 | H |
| *trnA* | 11774 | 11841 | 68 |  |  |  | TGC | 1060 | L |
| *trnW* | 11848 | 11916 | 69 |  |  |  | TCA | 6 | H |
| *trnM* | 11940 | 12008 | 69 |  |  |  | CAT | 23 | H |
| *trnG* | 12020 | 12085 | 66 |  |  |  | TCC | 11 | H |
| *trnP* | 12282 | 12350 | 69 |  |  |  | TGG | 196 | H |
| *trnQ* | 12347 | 12417 | 71 |  |  |  | TTG | -4 | L |
| *trnN* | 12418 | 12484 | 67 |  |  |  | GTT | 0 | H |
| *trnL_1_* | 12485 | 12556 | 72 |  |  |  | TAG | 0 | H |
| *trnC* | 12565 | 12630 | 66 |  |  |  | GCA | 8 | H |
| *trnV* | 12631 | 12700 | 70 |  |  |  | TAC | 0 | L |
| *trnD* | 12707 | 12773 | 67 |  |  |  | GTC | 6 | L |
| *trnY* | 12774 | 12842 | 69 |  |  |  | GTA | 0 | H |
| *trnL_2_* | 12866 | 12937 | 72 |  |  |  | TAA | 23 | H |
| *nad1* | 12941 | 13912 | 972 | 323 | ATG | TAA |  | 3 | H |
| *trnI* | 13924 | 13991 | 68 |  |  |  | GAT | 11 | H |
| *nad2* | 13992 | 15032 | 1041 | 346 | ATG | TAA |  | 0 | H |
| *trnT* | 15035 | 15104 | 70 |  |  |  | TGT | 2 | H |
| *16S* | 16115 | 17566 | 1452 |  |  |  |  | 1010 | H |
