# Supplementary material for: Complete mitochondrial genome of Benthodytes marianensis (Holothuroidea: Elasipodida: Psychropotidae): Insight into deep sea adaptation in the sea cucumber
Source: PLoS One. 2018 Nov 30;13(11):e0208051. doi: 10.1371/journal.pone.0208051 (PMC6267960; doi:10.1371/journal.pone.0208051)
Supplement: S5 Table — (DOCX) [file pone.0208051.s005.docx]

**Supplementary Table 5: Information concerning the echinoderm species with complete mitochondrial genome used in this study.**

|  |  | **Species** | **Accession Number** | **Classification** |
| --- | --- | --- | --- | --- |
| **Holothuroidea** | **1** | *Apostichopus japonicus* | NC_012616 | Holothuroidea; Aspidochirotacea; Synallactida; Stichopodidae |
|  | **2** | *Benthodytes marianensis* | MH208310 | Holothuroidea; Aspidochirotacea; Elasipodida; Psychropotidae |
|  | **3** | *Cucumaria miniata* | NC_005929 | Holothuroidea; Dendrochirotacea; Dendrochirotida; Cucumariidae |
|  | **4** | *Holothuria forskali* | NC_013884 | Holothuroidea; Aspidochirotacea; Holothuriida; Holothuriidae |
|  | **5** | *Holothuria scabra* | NC_027086 | Holothuroidea; Aspidochirotacea; Holothuriida; Holothuriidae |
|  | **6** | *Parastichopus californicus* | NC_026727 | Holothuroidea; Aspidochirotacea; Synallactida; Stichopodidae |
|  | **7** | *Parastichopus nigripunctatus* | NC_013432 | Holothuroidea; Aspidochirotacea; Synallactida; Stichopodidae |
|  | **8** | *Parastichopus parvimensis* | NC_029699 | Holothuroidea; Aspidochirotacea; Synallactida; Stichopodidae |
|  | **9** | *Peniagone sp. YYH-2013* | KF915304 | Holothuroidea; Aspidochirotacea; Elasipodida; Elpidiidae |
|  | **10** | *Stichopus horrens* | NC_014454 | Holothuroidea; Aspidochirotacea; Synallactida; Stichopodidae |
|  | **11** | *Stichopus sp. SF-2010* | NC_014452 | Holothuroidea; Aspidochirotacea; Synallactida; Stichopodidae |
| **Asteroidea** | **12** | *Acanthaster brevispinus* | NC_007789 | Asteroidea; Valvatacea; Valvatida; Acanthasteridae |
|  | **13** | *Acanthaster planci* | NC_007788 | Asteroidea; Valvatacea; Valvatida; Acanthasteridae |
|  | **14** | *Aphelasterias japonica* | NC_025766 | Asteroidea; Forcipulatacea; Forcipulatida; Asteriidae |
|  | **15** | *Asterias amurensis* | NC_006665 | Asteroidea; Forcipulatacea; Forcipulatida; Asteriidae |
|  | **16** | *Astropecten polyacanthus* | NC_006666 | Asteroidea; Valvatacea; Paxillosida; Astropectinidae |
|  | **17** | *Luidia quinaria* | NC_006664 | Asteroidea; Valvatacea; Paxillosida; Luidiidae |
|  | **18** | *Patiria pectinifera* | NC_001627 | Asteroidea; Valvatacea; Valvatida; Asterinidae |
| **Ophiuroidea** | **19** | *Amphipholis squamata* | NC_013876 | Ophiuroidea; Ophiuridea; Ophiurida; Ophiurina; Gnathophiurina;Amphiuridae |
|  | **20** | *Astrospartus mediterraneus* | NC_013878 | Ophiuroidea; Ophiuridea; Euryalida; Gorgonocephalidae |
|  | **21** | *Ophiacantha linea* | NC_023254 | Ophiuroidea; Ophiuridea; Ophiurida; Ophiurina; Ophiacanthidae |
|  | **22** | *Ophiocomina nigra* | NC_013874 | Ophiuroidea; Ophiuridea; Ophiurida; Ophiurina; Gnathophiurina; Ophiocomidae |
|  | **23** | *Ophiopholis aculeata* | NC_005334 | Ophiuroidea; Ophiuridea; Ophiurida; Ophiurina; Gnathophiurina; Ophiactidae |
|  | **24** | *Ophiura albida* | NC_010691 | Ophiuroidea; Ophiuridea; Ophiurida; Ophiurina; Chilophiurina; Ophiuridae; Ophiurinae |
|  | **25** | *Ophiura lutkeni* | NC_005930 | Ophiuroidea; Ophiuridea; Ophiurida; Ophiurina; Chilophiurina; Ophiuridae; Ophiurinae |
| **Crinoidea** | **26** | *Antedon mediterranea* | NC_010692 | Crinoidea; Articulata; Comatulida; Antedonidae |
|  | **27** | *Florometra serratissima* | NC_001878 | Crinoidea; Articulata; Comatulida; Antedonidae |
|  | **28** | *Neogymnocrinus richeri* | NC_007689 | Crinoidea; Articulata; Cyrtocrinida; Sclerocrinidae |
|  | **29** | *Phanogenia gracilis* | NC_007690 | Crinoidea; Articulata; Comatulida; Comatulidae; Comatulinae |
| **Echinoidea** | **30** | *Arbacia lixula* | NC_001770 | Echinoidea; Euechinoidea; Echinacea; Arbacoida; Arbaciidae |
|  | **31** | *Diadema setosum* | NC_033522 | Echinoidea; Euechinoidea; Diadematacea; Diadematoida; Diadematidae |
|  | **32** | *Echinocardium cordatum* | NC_013881 | Echinoidea; Euechinoidea; Atelostomata; Spatangoida; Loveniidae |
|  | **33** | *Echinometra mathaei* | NC_034767 | Echinoidea; Euechinoidea; Echinacea; Echinoida; Echinometridae |
|  | **34** | *Echinothrix diadema* | NC_033523 | Echinoidea; Euechinoidea; Diadematacea; Diadematoida; Diadematidae |
|  | **35** | *Glyptocidaris crenularis* | NC_032365 | Echinoidea; Euechinoidea; Diadematacea; Phymosomatoida; Phymosomatidae |
|  | **36** | *Heliocidaris crassispina* | NC_023774 | Echinoidea; Euechinoidea; Echinacea; Echinoida; Echinometridae |
|  | **37** | *Hemicentrotus pulcherrimus* | NC_023771 | Echinoidea; Euechinoidea; Echinacea; Echinoida; Strongylocentrotidae |
|  | **38** | *Heterocentrotus mammillatus* | NC_034768 | Echinoidea; Euechinoidea; Echinacea; Echinoida; Echinometridae |
|  | **39** | *Loxechinus albus* | NC_023770 | Echinoidea; Euechinoidea; Echinacea; Echinoida; Echinidae |
|  | **40** | *Mesocentrotus franciscanus* | NC_024177 | Echinoidea; Euechinoidea; Echinacea; Echinoida; Strongylocentrotidae |
|  | **41** | *Mesocentrotus nudus* | NC_020771 | Echinoidea; Euechinoidea; Echinacea; Echinoida; Strongylocentrotidae |
|  | **42** | *Mespilia globulus* | NC_034769 | Echinoidea; Euechinoidea; Echinacea; Temnopleuroida; Temnopleuridae |
|  | **43** | *Nacospatangus alta* | NC_023255 | Echinoidea; Euechinoidea; Atelostomata; Spatangoida; Maretiidae |
|  | **44** | *Paracentrotus lividus* | NC_001572 | Echinoidea; Euechinoidea; Echinacea; Echinoida; Echinidae |
|  | **45** | *Pseudocentrotus depressus* | NC_023773 | Echinoidea; Euechinoidea; Echinacea; Echinoida; Echinidae |
|  | **46** | *Salmacis bicolor* | KU302104 | Echinoidea; Euechinoidea; Echinacea; Temnopleuroida; Temnopleuridae |
|  | **47** | *Salmacis sphaeroides* | NC_033528 | Echinoidea; Euechinoidea; Echinacea; Temnopleuroida; Temnopleuridae |
|  | **48** | *Sterechinus neumayeri* | KJ680295 | Echinoidea; Euechinoidea; Echinacea; Echinoida; Echinidae |
|  | **49** | *Strongylocentrotus droebachiensis* | NC_009940 | Echinoidea; Euechinoidea; Echinacea; Echinoida; Strongylocentrotidae |
|  | **50** | *Strongylocentrotus intermedius* | KY964300 | Echinoidea; Euechinoidea; Echinacea; Echinoida; Strongylocentrotidae |
|  | **51** | *Strongylocentrotus pallidus* | NC_009941 | Echinoidea; Euechinoidea; Echinacea; Echinoida; Strongylocentrotidae |
|  | **52** | *Strongylocentrotus purpuratus* | NC_001453 | Echinoidea; Euechinoidea; Echinacea; Echinoida; Strongylocentrotidae |
|  | **53** | *Temnopleurus hardwickii* | NC_026200 | Echinoidea; Euechinoidea; Echinacea; Temnopleuroida; Temnopleuridae |
|  | **54** | *Temnopleurus reevesii* | NC_033530 | Echinoidea; Euechinoidea; Echinacea; Temnopleuroida; Temnopleuridae |
|  | **55** | *Temnopleurus toreumaticus* | NC_033529 | Echinoidea; Euechinoidea; Echinacea; Temnopleuroida; Temnopleuridae |
|  | **56** | *Tripneustes gratilla* | NC_034770 | Echinoidea; Euechinoidea; Echinacea; Temnopleuroida; Toxopneustidae |
